# Nanomaterials for the Selective Detection of Hydrogen Sulfide in Air

**DOI:** 10.3390/s17020391

**Published:** 2017-02-17

**Authors:** Eduard Llobet, Jérôme Brunet, Alain Pauly, Amadou Ndiaye, Christelle Varenne

**Affiliations:** 1 MINOS-EMaS, Universitat Rovira i Virgili, E-43007 Tarragona, Spain; 2 CNRS, Institut Pascal, Université Clermont Auvergne, F-63000 Clermont-Ferrand, France; jerome.brunet@univ-bpclermont.fr (J.B.); alain.pauly@lasmea.univ-bpclermont.fr (A.P.); amadou.ndiaye@univ-bpclermont.fr (A.N.); christelle.VARENNE@lasmea.univ-bpclermont.fr (C.V.)

**Keywords:** hydrogen sulfide, gas sensor, nanomaterials, air quality monitoring

## Abstract

This paper presents a focused review on the nanomaterials and associated transduction schemes that have been developed for the selective detection of hydrogen sulfide. It presents a quite comprehensive overview of the latest developments, briefly discusses the hydrogen sulfide detection mechanisms, identifying the reasons for the selectivity (or lack of) observed experimentally. It critically reviews performance, shortcomings, and identifies missing or overlooked important aspects. It identifies the most mature/promising materials and approaches for achieving inexpensive hydrogen sulfide sensors that could be employed in widespread, miniaturized, and inexpensive detectors and, suggests what research should be undertaken for ensuring that requirements are met.

## 1. Introduction

Hydrogen sulfide is a gas included in the list of toxic and reactive highly hazardous chemicals from the Occupational Safety and Health Administration (OSHA) [1]. A concentration of 100 ppm of H_2_S is considered to be immediately dangerous to life and health (IDLH). Since H_2_S occurs in crude petroleum, natural gas, and hot springs, the main activities in which occupational exposure is likely are petroleum and natural gas drilling, refining, and coke ovens [2,3]. Additionally, since hydrogen sulfide is formed during the decay of organic matter, wastewater treatment plants, landfills, and tanneries are also important emitting sources [4]. Finally, the Kraft process employed in many paper mills, which involves using sodium hydroxide and sodium sulfide also results in the emission of H_2_S.

Besides the odor nuisance often resulting from hydrogen sulfide emissions, human toxicology data is available, compiled from acute poisoning case reports and occupational exposures. Reported health effects in humans following exposure to hydrogen sulfide include death and respiratory, ocular, neurological, cardiovascular, metabolic, and reproductive effects. The lowest-observed-adverse-effect level (LOAEL) is as low as 2.8 mg/m^3^ (1.87 ppm) in asthmatic individuals for respiratory and neurological effects [5,6].

The analytical methods most commonly used to measure hydrogen sulfide in air include gas chromatography (GC) or the spectrophotometric method. The latter involves a fixation step in which H_2_S gas is trapped in a solution that is further made to react with formaldehyde forming a colored product, the absorbance of which is related to hydrogen sulfide concentration [7]. Detection limits reported for the analysis of hydrogen sulfide in air are 10 mg/m^3^ (6.7 ppm) for GC employing a flame ionization detector, or 0.2 mg/m^3^ (130 ppb) in spectrophotometry [5]. However, these methods are rather expensive, cumbersome, and not suited for implementing a widespread, continuous monitoring of hydrogen sulfide in ambient conditions. Nowadays, most commercially available hydrogen sulfide detectors employ either electrochemical or semiconductor gas sensors. These have a lower detection limit of about 1 ppm, they are affected by ambient humidity, their power consumption is rather high, and they require frequent recalibrations, especially when integrated in portable equipment. Electrochemical sensors employing a 3-electrode configuration and liquid electrolyte can be a good, inexpensive approach for detecting hydrogen sulfide in air at concentrations up to thousands of ppm with ppm resolution [8]. The typical cost of an electrochemical hydrogen sulfide sensor is about 30 US dollars. Selectivity can be achieved by the proper selection of the materials’ integrating filter, hydrophobic membrane, working electrode and electrolyte [9,10]. However, their internal architecture makes them very hard to miniaturize.

There is a strong demand for miniaturized, inexpensive sensors to be integrated in portable/wearable devices for personal protection or in distributed wireless sensor networks for environmental monitoring. In the last few years, there have been considerable research efforts devoted to the development of inorganic, organic, and hybrid nanomaterials in view of achieving hydrogen sulfide sensors with improved sensitivity, selectivity, resilience to ambient humidity, long-term stability, and low power consumption. The object of this review paper is to identify these recent efforts, summarize achievements, identify shortcomings, and discuss future research directions.

## 2. Metal Oxide Nanomaterials

Even though metal oxide nanomaterials are appropriate for building inexpensive and sensitive enough hydrogen sulfide sensors, they generally suffer from a lack of selectivity, are heavily affected by ambient moisture, and are power-hungry, since their optimal operating temperatures are well above room temperature. Despite these initial drawbacks, many studies have been conducted on metal oxide hydrogen sulfide sensors. The following paragraphs discuss the main results attained thus far.

Iron oxide (Fe_2_O_3_) could be anticipated to be a good candidate for detecting H_2_S, because the material had been widely employed as a catalyst in the oxidation of hydrogen sulfide in desulfurization processes [11]. Indeed, Zhang and co-workers [12] demonstrated that when H_2_S was catalytically oxidized in Fe_2_O_3_, an intense chemiluminescent (CL) signal at 400 nm could be recorded. Furthermore, the CL signal was found to change linearly with hydrogen sulfide in a wide concentration range. The CL activity of Fe_2_O_3_ in the presence of H_2_S was found to be at least one order of magnitude higher than that of other catalysts such as CaO, Al_2_O_3_, ZrO_2_, or Au-loaded WO_3_. In addition, Fe_2_O_3_ was very selective towards the detection of hydrogen sulfide with no cross-sensitivity to ethanol, hexane, cyclohexane, ethylene, hydrogen, o-dichlorobenzene, carbon dioxide, nitrogen dioxide, ammonia, thiophene, and sulfur dioxide. Even though it showed a CL response to dimethyl sulfur, this happened at operating temperatures higher than 360 °C, therefore, this potential cross-sensitivity could be addressed by operating Fe_2_O_3_ at 320 °C. The reason for such good performance in terms of selectivity was attributed to Fe_2_O_3_ being able to produce CL intermediates (excited SO_2_) upon the catalytic oxidation of H_2_S. The system was studied under dry conditions only and, therefore, the influence of ambient moisture in overall performance was not discussed. A similar CL behavior was observed by Liu and co-workers [13] on self-assembled SnO_2_ nanospheres, synthesized by a hydrothermal method. In this particular case, changes in the CL signal at 410 nm were recorded in the presence of H_2_S at near 200 ppb levels. However, no selectivity study was conducted.

**Figure 1:**
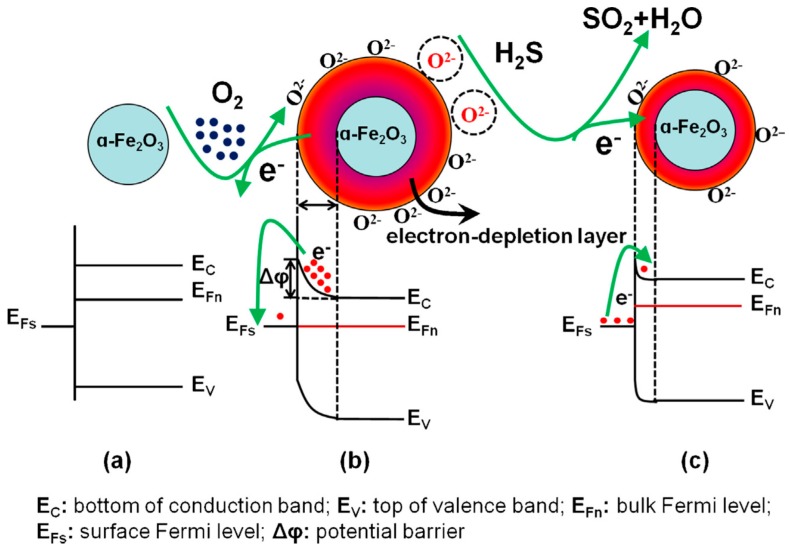
Band diagrams and schematic images of the surface reactions of an α-Fe_2_O_3_ nanograin under different atmospheres, while kept at the optimal operating temperature (i.e., 300 °C): (**a**) under inert atmosphere (e.g., Ar); (**b**) exposed to air (**c**) in the presence of H_2_S diluted in air. Reproduced from [15], with permission from Elsevier.

Besides its interesting chemiluminescent properties, iron oxide has also been employed as the active material in chemo-resistors for detecting hydrogen sulfide. Zheng and co-workers [14] reported the ionothermal synthesis of α-Fe_2_O_3_ nanochains and its use in resistive hydrogen sulfide sensors. Similarly, Li and co-workers [15] employed α-Fe_2_O_3_ nanoparticles for detecting H_2_S at the ppm level. The sensing mechanism consists of H_2_S altering the equilibrium concentration of oxygen adsorbates present at the surface of the iron oxide nanomaterial. At the operating temperature (i.e., near 300 °C) H_2_S reacts with oxygen adsorbates producing SO_2_ and H_2_O. H_2_S oxidation lowers the amount of ionosorbed oxygen at the surface of Fe_2_O_3_. This increases the number of charge carriers (electrons) in the conduction band of the nanomaterial (an *n*-type semiconductor) and lowers the width of the space charge layer that develops, upon oxygen ionosorption, at the outer shell of the Fe_2_O_3_ nanostructures. Macroscopically, this translates to a decrease in the electrical resistance of a film made of the nanomaterial. This detection is reversible because when the material is flushed with clean air, the initial concentration of ionosorbed oxygen is restored, and the baseline resistance is regained. Figure 1 illustrates this detection mechanism. Even though this nanomaterial is highly sensitive to hydrogen sulfide given its high catalytic properties for H_2_S oxidation, its detection mechanism implies an inherent lack of selectivity, since the presence of any reducing species able to affect the equilibrium concentration of oxygen adsorbates, would generate a confounding signal.

In view of ameliorating the selectivity for detecting H_2_S, the metal loading of metal oxides and the use of spinels has been reported. For example, Wu and co-workers [16] reported the use of Pt-loaded α-Fe_2_O_3_. The presence of Pt clusters on the surface of α-Fe_2_O_3_ results in an increased concentration of oxygen adsorbates on the metal oxide. Pt adsorbs and decomposes molecular oxygen, which eventually ionosorbs on the surface of the metal oxide via a spill-over effect [17]. The increased amount of oxygen adsorbates justifies the increased response to H_2_S that is observed experimentally. However, this mechanism is not able to explain why there should be any selectivity improvement. Mulla and co-workers [18] employed Fe-doped SnO_2_. At the reported operating temperatures, iron nanoparticles are most probably oxidized and these act as sensitizers for the detection of hydrogen sulfide. However, the selectivity attained remains poor. Zhu and co-workers [19] reported the use of porous iron molybdate nanorods. A good response to hydrogen sulfide was obtained at a remarkably low operating temperature (80 °C), but no details on selectivity or ambient humidity effects were given. The use of *p*-type semiconductor spinels such as nickel ferrite (NiFe_2_O_4_) or Ni_0.6_Zn_0.4_Fe_2_O_4_ has been reported as well [20,21]. However, their sensitivity to H_2_S is significantly lower than that of the materials discussed above and their selectivity remains limited.

Besides iron-containing nanomaterials, many other metal oxides have been investigated for developing hydrogen sulfide chemo-resistors. These include WO_3_ [22,23,24,25], ZnO [26,27,28], In_2_O_3_ [29], CeO_2_ [30], and YMnO_3_ [31]. Pure tungsten oxide shows a response to hydrogen sulfide (R_air_/R_gas_ = 5 at 225 °C for 20 ppm H_2_S) but also very high cross-sensitivity to ammonia [32] (R_air_/R_gas_ = 3.5 at 300 °C for 100 ppm NH_3_) and to nitrogen dioxide [22] (given the oxidizing nature of nitrogen dioxide, the sensor resistance increases when exposed to this species; R_gas_/R_air_ = 8 at 225 °C for 1 ppm NO_2_). Deng and co-workers [33] have reported a high response and a remarkable selectivity towards hydrogen sulfide of mesoporous tungsten trioxide having large pore widths, but the reason for this selectivity is not discussed and cannot be easily explained based on the size of the pores within their nanomaterial. Zinc oxide nanomaterials (nanorods and dendritic) [26,27] or ceria nanowires [30] have been shown to be responsive towards hydrogen sulfide at room temperature, an interesting result for developing ultra-low power sensors; however, they show heavy cross-sensitivity to ambient humidity and rather slow response dynamics. Indium oxide shows poor selectivity to hydrogen sulfide with important nitrogen dioxide [34] and ethanol [29] cross-sensitivity. Unlike the previously discussed materials, hexagonal YMnO_3_ is a *p*-type semiconductor. It shows moderate sensitivity to hydrogen sulfide with limited selectivity.

**Figure 2:**
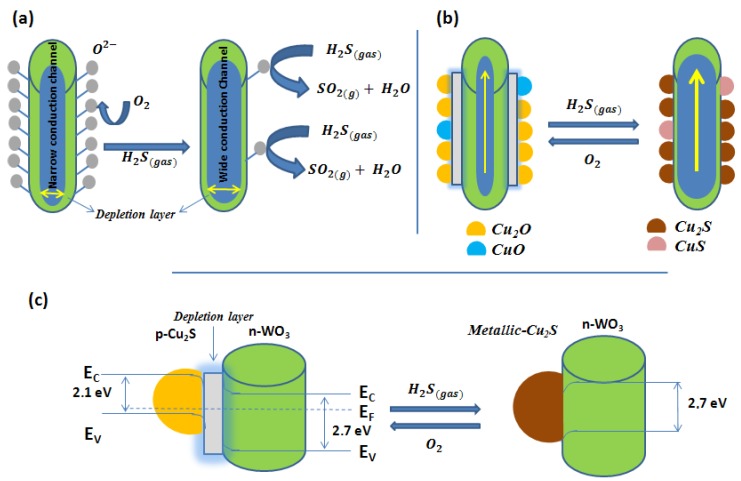
H_2_S gas sensing mechanism of (**a**) pure WO_3_ nanowires, (**b**) Cu_2_O/CuO nanoparticle-functionalized WO_3_ nanowires, and (**c**) an example of the evolution of the Cu_2_O nanoparticle/WO_3_ nanowire p-n heterojunction before and after exposure to H_2_S. Reproduced from [47], with permission from the American Chemical Society.

In 1994, Rao and co-workers [35] developed a H_2_S sensor employing CuO nanoparticles dispersed into a SnO_2_ matrix. The transformation of CuO, a *p*-type semiconductor, into CuS, which shows metallic characteristics, upon exposure to H_2_S is hypothesized to be the reason for the large increase observed in the conductance of the CuO-SnO_2_ film. Furthermore, at the sensor operating temperature (200 °C) CuS converts back to CuO when H_2_S is removed from the ambient conditions, which results in the reversibility of the detection mechanism. In 1998, Yamazoe and co-workers [36] and Gaskow and co-workers [37] explored further the use of CuO-SnO_2_ heterojunctions for the detection of H_2_S. Gaskov and coworkers [38] attributed the detection mechanism to the transformation of CuO into Cu_2_S, as observed by X-ray diffraction (XRD) analysis. The selectivity in the detection of hydrogen sulfide and the effect of ambient moisture are not reported in these initial studies. Since then, many authors have reported the use of *p*-type cuprous or cupric oxides onto *n*-type metal oxides for detecting H_2_S. These include CuO-SnO_2_ [38,39,40,41,42], Cu_2_O-SnO_2_ [43], CuO-ZnO [44,45], CuO-SnO_2_-ZnO [46], and Cu_2_O-WO_3_ [47] nanocomposites. Most papers show that the optimal operating temperature lies in the range from 100 °C to 400 °C, however, a few examples are given on the room-temperature detection of hydrogen sulfide [40,43]. In room-temperature operated sensors, some problems arise related to the slow response dynamics and the lack of recovery of the sensor baseline. While in most cases the response of the nanomaterials to hydrogen sulfide is only discussed, some authors properly address selectivity issues by studying the sensor response to possible interfering species [41,47], including the effect of ambient humidity [48]. According to these results, there seems to be an optimum in the amount of copper oxide loading, which lies between 0.5% and 3%, for maximizing selectivity towards H_2_S and minimizing cross-sensitivity to changes in the level of the ambient moisture. In many cases, the response towards H_2_S is found to be, at least, over 10-fold higher than that observed for the other species tested (e.g., CH_4_, CO, NH_3_, H_2_, C_6_H_6_, or NO_2_). Figure 2 illustrates the gas sensing mechanism.

As shown in Figure 2a, when pure WO_3_ nanowires are exposed to air, oxygen molecules can adsorb on their surface and form chemisorbed oxygen species. Oxygen adsorbates lead to the formation of an electron depletion layer by trapping electrons via the conduction band of the *n*-type tungsten oxide and make the material highly resistive. When exposed to H_2_S, the chemisorbed oxygen species react with the reducing gas molecules producing H_2_O and SO_2_, while the electrons, originally trapped at oxygen adsorbates, are released. This results in a decrease in the resistance of the nanowires. This mechanism, which is equivalent to the one illustrated in Figure 1, is not selective, since any reducing gas could, in principle, alter the concentration of oxygen adsorbates and, therefore, would be detected. In contrast, copper oxide decorated WO_3_ nanowires show a different mechanism (Figure 2b,c). Copper oxide nanoparticles and WO_3_ nanowires are *p*-type and *n*-type, respectively. The contact between these two different materials leads to the formation of numerous p-n heterojunctions and electron depletion layers at their interface. Upon exposure to H_2_S, copper oxides are converted to metallic copper sulfides, by an oxygen/sulfur replacement mechanism, and the p-n heterojunctions are destroyed. Hence, a large number of electrons are released in the WO_3_ nanowires and a dramatic decrease in the resistance of the nanocomposite can be observed. In the recovery phase, when hydrogen sulfide is removed by a flow of clean air, the copper oxides are regenerated, the p-n heterojunctions are restored, and the material resistance returns to its original high value. This mechanism, which has been experimentally validated by performing X-ray photoelectron spectroscopy (XPS) analysis on the nanomaterials before and after their exposure to hydrogen sulfide [47], explains why copper oxide decorated metal oxide composites show an inherent selectivity to hydrogen sulfide.

Besides the use of p-n heterojunctions formed by copper oxide nanoparticles supported on an *n*-type metal oxide semiconductor, some authors have reported the use of pure copper oxide nanomaterials, e.g., CuO nanosheets [48] or thin films with different stoichiometry (CuO, Cu_2_O, or Cu_4_O_3_) [49,50]. The results reported are also very promising for the selective detection of H_2_S, however, the response of pure copper oxide nanomaterials seems to be affected by ambient moisture [48,50]. In contrast, some heterojunction nanomaterials have been reported to be humidity-insensitive [47]. This synergistic effect found in p-n heterojunction nanomaterials deserves further research to be better understood.

Even though the vast majority of papers in which the use of metal oxide nanomaterials is addressed for detecting hydrogen sulfide employ chemo-resistive transducing schemes, a few papers have reported optical transduction via the monitoring of changes in surface plasmon resonance (SPR). Martucci and co-workers [51] reported Au nanoparticles dispersed onto TiO_2_-NiO films for detecting H_2_S. The direct oxidation of H_2_S on the films resulting in SO_2_ is described as the sensing mechanism. The response signal is the change in absorbance intensity at a wavelength that is close to the SPR of Au nanoparticles (600 nm). Despite this, the nanomaterial seems selective to hydrogen sulfide in the presence of CO or H_2_, the detection limit is in the ppm range, and the signal saturates at H_2_S concentrations of about 5 to 10 ppm. Gupta and co-workers [52] reported positive shifts in the SPR of Cu-ZnO films with increasing concentrations of H_2_S (10 to 100 ppm). The mechanism of detection is not discussed, but most probably the shift observed is due to the sulfurization of Cu upon exposure to H_2_S. In a very similar approach, the same group reported SPR in nickel oxide doped indium tin oxide (ITO) films over silver [53], and in ZnO nanoparticles or nanorods [54,55]. Positive shifts in the SPR of these films with increasing concentrations of H_2_S are observed. This is attributed to the formation of NiS or ZnS upon exposure to H_2_S. No significant cross-sensitivity towards ammonia, chlorine, or carbon monoxide is reported. A saturation of the SPR response at about 80 to 100 ppm is observed.

## 3. Functionalized Carbon Nanomaterials

In 2009, Mhaisalkar and co-workers [56] described the use of Ag decorated single walled carbon nanotubes (SWCNT) for detecting H_2_S at room temperature in a background of nitrogen. These resistive sensors show an irreversible response towards H_2_S, due to the formation of Ag_2_S. Furthermore, the sensor is not selective, since it shows cross-sensitivity to nitric oxide and carbon monoxide. In 2010, Deshusses and co-workers [57] employed Au-decorated SWCNTs for detecting H_2_S at room temperature in air. The detection of H_2_S at a concentration as low as 20 ppb was shown. Operated as resistive films on a back gate configuration, the affinity between gold and sulfur and the fact that the work function of Au changes upon the chemisorption of H_2_S is hypothesized as the sensing mechanism. When proper back gate voltages are applied, the sensor signal is reversible, which is attributed to mild heating of the Au-SWCNT mat. However, it is well-known that Au-decorated carbon nanotubes are very sensitive to other gaseous species such as NO_2_ [58] and no selectivity studies were performed. More recently, Yoon and co-workers [59] reported the use of a Co_3_O_4_-SWCNT composite for detecting H_2_S at 250 °C. The role of the small amount of carbon nanotubes within the nanocomposite is to render the cobalt oxide nanoparticles more defective and, thus, more sensitive to hydrogen sulfide. A selectivity study was performed in which small cross-sensitivity to ammonia, methane, and hydrogen was reported. Besides the use of carbon nanomaterials in resistive H_2_S sensors, Cu-decorated SWCNTs have been employed together with gravimetric transducers [60]. The sensor is responsive to H_2_S at ppm levels and no significant cross-sensitivity to hydrogen, ethanol or acetone is reported. The effect of ambient moisture on sensor response is also discussed. A 40% relative humidity (R.H.) level completely destroys room temperature sensitivity. To recover the response signal in the presence of humidity, the device has to be operated at temperatures well above 100 °C.

Pal and co-workers [61] reported the potential of using a composite consisting of a tin oxide thin film coated with diamond like carbons (DLC) in which Cu nanoparticles had been embedded. It was observed that soon after exposure to hydrogen sulfide (about 3 s), the characteristic surface plasmon resonance peak, located at 650 nm, disappeared. This was attributed, via XRD and Raman studies to the formation of Cu_x_S during the exposure to H_2_S. These results are very preliminary, since a rather high concentration of hydrogen sulfide was tested (i.e., 1000 ppm) and the dynamic range of the response, reversibility, selectivity, and stability of the detector were not investigated.

**Figure 3:**
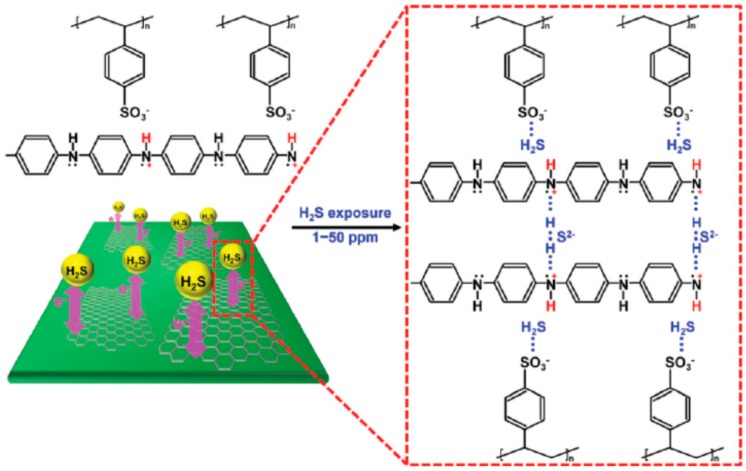
Possible mechanism for molecular interactions between the PSS-doped PANI/graphene nanocomposites and H_2_S gas molecules. Reproduced from [64], with permission from the Royal Society of Chemistry.

In the last few years, the use of graphene and graphene-related materials and composites has gained attention for the detection of hydrogen sulfide. Chen and co-workers [62] described the use of Cu_2_O onto functionalized graphene sheets (FGS). FGS acts as a molecular template for the controlled nucleation of Cu_2_O nanoparticles, the size of which is controlled via the C/O ratio in the FGS. Upon adsorption of hydrogen sulfide, Cu_2_O nanoparticles inject electrons onto the p-type FGS, which results in an increase in the resistance of the nanocomposite film. The detection of hydrogen sulfide down to 5 ppb, and operating the sensors at room temperature is demonstrated. Selectivity is also described, since small responses to hydrogen, ammonia, ethanol and methane were reported, but no information is given on cross-sensitivity to ambient moisture or nitrogen dioxide. In a similar approach, Jahangiri and co-workers [63] have reported MoO_3_ on reduced graphene oxide (MoO_3_/rGO). However, the optimal operating temperature was 160 °C and the limit of detection for H_2_S was rather high, near 50 ppm. Jang and co-workers [64], reported the use of a conducting polymer/graphene nanocomposite, namely poly(4-styrenesulfonic acid)-doped polyaniline/graphene (PSS-doped PANI/graphene) operated at room temperature. PSS was used as a doping and a binding agent for the polymerization of aniline monomers. The PSS allowed the dispersion of reduced graphene sheets through electrostatic repulsion. PSS-doped PANI/graphene composites containing 30 wt% graphene showed the highest response to hydrogen sulfide at ppm levels. As sensing mechanisms, the formation of additional N–H bonds that appear in the PSS-doped PANI when in the presence of hydrogen sulfide is suggested. This would result in the experimentally observed decrease in the resistance of the PSS-doped PANI structure. Figure 3 illustrates this mechanism. The dispersed graphene sheets allow higher electric currents to flow through the sensor electrode, which significantly increases the response and lowers the detection limit for hydrogen sulfide. This composite, however, shows an important cross-sensitivity to ammonia.

Recently, the use of amide or amine functionalized graphene oxide has been reported for the detection of hydrogen sulfide at room temperature [65,66]. However, the response is rather weak and subject to strong cross-sensitivity issues.

## 4. Other Nanomaterials and Transduction Approaches

In 2013, Xie and co-workers [67] reported organic thin film transistors employing silicon dioxide as a dielectric layer and copper phthalocyanines as gas sensitive materials for detecting hydrogen sulfide. The detection of hydrogen sulfide was demonstrated at room temperature in the hundreds of ppm range, with some reversibility problems. These sensors suffered from important cross-sensitivity to SO_2_ and the effect of ambient moisture was not reported. Furthermore, Cu-phthalocyanine sensors for detecting ethanol, nitrogen dioxide, water vapor, or volatile organic compounds have been reported [68,69], so important selectivity issues can be anticipated.

Liu and co-workers [70] reported organic-inorganic composites based on polythiophene (PT), a p-type semiconductor, and WO_3_ (*n*-type) for detecting H_2_S at low operating temperatures (70 °C). The changes observed in the electrical resistance of the film are attributed to the decrease in the width of the space charge region that develops at the PT-WO_3_ interface, when exposed to hydrogen sulfide. These sensors show low cross-sensitivity to methanol, ethanol, propanol, and ammonia, however, no data on humidity interference is shown.

Recently, Tang and co-workers [71,72] have shown that colloidal PbS quantum dots (QDs) show remarkable sensitivity and selectivity to H_2_S when operated at 135 °C. A 4000-fold decrease in resistance is reported when the sensor is exposed to 50 ppm of hydrogen sulfide. The originally oleic-acid caped PbS QDs were treated with Pb(NO_3_)_2_ for ligand exchange. The gas sensing mechanism may be twofold: the interaction with hydrogen sulfide causes the removal of adsorbed oxygen on the surface of the QDs, which change from p-type to n-type. Additionally, H_2_S adsorption creates donor states near the conduction band. As a result of these two combined effects, a dramatic change in resistance is observed. Furthermore, a remarkable selectivity towards H_2_S is shown, with little cross-sensitivity to SO_2_, NO_2_, and NH_3_. Once more, measurements were performed under dry conditions only and no information on ambient moisture effects was shown. This new approach seems very promising, yet the results reported so far are somewhat preliminary. More efforts are needed to better understand and model the sensing mechanisms for the technique to increase in maturity.

Metal-organic frameworks (MOFs) are built from clusters of metal ions and multidentate organic ligands. MOFs offer a wide spectrum of structures, pore sizes, and metal catalytic sites, together with high specific area to interact with gases and good thermal stability. In the last few years, MOFs have attracted interest for designing gas sensors, including H_2_S sensors [73]. In this paper, different MOFs were assessed for the detection of H_2_S at ppm levels, and Zn_3_(BTC)_2_·12H_2_O or ZIF-8 showed a remarkably intense and selective cataluminescence (CL) response at 250 °C. According to the results, the nature of the metal sites is more important than the ligands within the MOFs for producing a CL signal, even though the latter modulate the intensity of this response. Extraordinary fast response and recovery times below 1 s and 5 s, respectively, have been reported. Furthermore, MOFs could also be employed as functional coatings onto metal oxide nanomaterials, as reported in [74]. Acting as molecular sieves, MOFs could be of help for improving selectivity.

**Figure 4:**
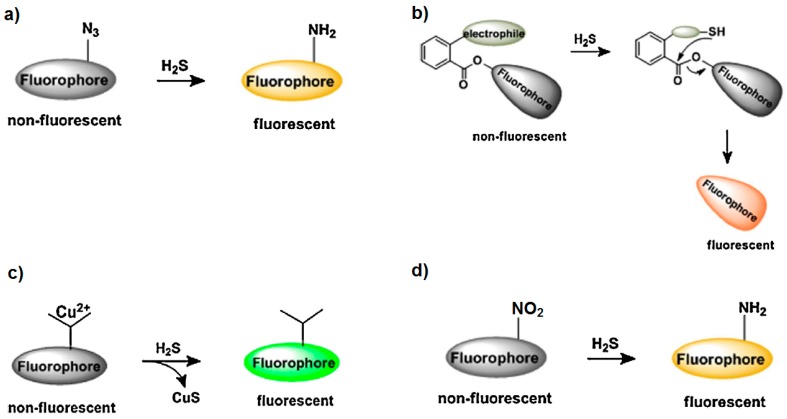
Reactions for the selective detection of H_2_S employing fluorescent probes. Reduction of azides into amines by H_2_S (**a**), nucleophilic addition of H_2_S to the probe resulting in a cyclization process that generates a fluorescent molecule (**b**), H_2_S mediates the precipitation of CuS (**c**), nitro to amine reduction (**d**). Adapted from [75], with permission from Elsevier.

The selective detection of H_2_S employing fluorescent probes has also been reported. Fluorescent probes for detecting hydrogen sulfide employ four different reactions to produce an easily readable, turn-on fluorescence [75]. These reactions consist of the selective reduction of azides into amines by H_2_S; the nucleophilic addition of H_2_S to the probe which results in a cyclization process that generates a fluorescent molecule; copper displacement in which H_2_S mediates precipitation of CuS to induce turn-on fluorescence; and nitro to amine reduction, in which H_2_S selectively reduces the nitro group into amine, producing turn-on of the fluorescence. Figure 4 gives a schematic overview of these four different approaches. Although this approach could be used to assess H_2_S in the environment, fluorescent probes have been specifically developed for selectively detecting H_2_S emitted by cells in living organisms as a biomarker of different diseases including cancer or Alzheimer’s disease [76,77].

## 5. Discussion

Considering the transducing schemes employed for detecting H_2_S, a significant majority of what has been reported discusses chemo-resistors. However, other approaches comprise chemo-field effect transistors (chemFET), gravimetric surface acoustic wave (SAW) or optical read-out via chemo-luminescence, monitoring of shifts in SPR, lossy mode resonance or absorbance changes in optical fiber sensors, and fluorescence (for detecting H_2_S in liquids). The sensitive materials reported include inorganic (metal oxides), carbon nanomaterials (carbon nanotubes, graphene-related, and diamond like, organic materials (phthalocyanines, conducting polymers) and organic-inorganic hybrid nanomaterials. Table 1 summarizes the properties and the main performance characteristics of the different nanomaterials and transducing schemes that have been reported in the detection of hydrogen sulfide.

Considering the results summarized in Table 1, the most promising approaches are as follows.

Employing α-Fe_2_O_3_ at temperatures near 300 °C, which presents strong chemiluminescence upon interaction with hydrogen sulfide at 400 nm. Very remarkable selectivity is achieved because α-Fe_2_O_3_ is able to produce CL intermediates (excited SO_2_) upon the catalytic oxidation of H_2_S. Furthermore, this mechanism enables measuring H_2_S in a wide concentration range (up to thousands of ppm) and tens of seconds and minutes have been reported as response and recovery times, respectively [12]. The process for obtaining α-Fe_2_O_3_ is standard and can be easily scaled up for mass production. It involves the precipitation of ferric hydroxide employing an aqueous solution of FeCl_3_ with ammonia, which is washed in water and further oxidized by annealing at 600 °C for 3 h. Despite all these interesting characteristics, studying the effect of ambient moisture on the sensor response and the long-term stability of the material would be necessary to fully validate this approach. In addition, monitoring CL at 400 nm involves using a chemiluminescence analyzer and optical filters, which are not easy to miniaturize and may be rather expensive.

Heterojunction nanomaterials that comprise *p*-type CuO or Cu_2_O and an *n*-type metal oxide such as SnO_2_, ZnO, or WO_3_. The detection mechanism consists of the strong electronic sensitization brought about by the copper oxides, which sulfurize to CuS or Cu_2_S upon exposure to H_2_S. In contrast to copper oxides, which are *p*-type semiconductors, copper sulfides are metallic and, therefore, exposure to H_2_S results in a dramatic increase in conductivity of the p-n nanomaterial. Furthermore, this sulfurization process is reversible, i.e., copper oxides are regenerated upon cleaning with air, which makes this approach very interesting for the chemoresistive sensing of H_2_S. Employing this approach, the detection of hydrogen sulfide in a wide concentration range has been reported (200 ppb to 1500 ppm) with a lower detection limit of about 20 ppb [35,36,37,38,39,40,41,42,43,44,45,46,47,52]. The best response and recovery times reported are units and tens of seconds, respectively. The optimal operating temperature lies between 140 °C and 400 °C, however, Gupta and co-workers [55] reported a remarkable response towards H_2_S despite sensors being operated at room temperature. Even though metal oxides are known to be poorly selective, the detection of hydrogen sulfide is a special niche in which copper oxide based nanomaterials can reach remarkable selectivity, due to the specific detection mechanism discussed above. Overall, these sensors do not present significant cross-sensitivity to many species such as hydrogen, carbon monoxide, liquefied petroleum gas, sulfur dioxide, ethanol, acetaldehyde, formaldehyde, toluene, benzene, ammonia, nitric oxide, nitrogen dioxide, methane, or acetone. While the effect of ambient moisture on responsiveness to hydrogen sulfide is almost never reported, Llobet and co-workers show in [47] that changes in background humidity only mildly affect H_2_S response. The processes employed for obtaining these nanomaterials are very different and eventually lead to strong dispersion in the performance characteristics of the sensors. Hydrothermal synthesis, precipitation formed salt precursors, and subsequent annealing or different types of chemical vapor deposition have been reported as synthesis methods, to name a few. The reported response intensities towards hydrogen sulfide in copper oxide supported on n-type metal oxides lie in a very wide range. The different synthesis methods employed result in materials with very different microstructures, namely, crystallite size, film porosity, and thickness. Considering what has been summarized in Table 1, higher response intensities are reported for thin films consisting of highly-porous materials with small crystallite sizes [37,38,39] or porous nanomaterials (nanorods, nanowires, or nanoribbons) having small diameters and supporting homogeneously-distributed, low-diameter copper oxide nanoparticles [40,42,44]. In principle, those synthesis methods that enable the procurement of nanomaterials with well controlled crystallinity (e.g., single crystalline) and nanodimensions (e.g., particles, wires, rods) would help increase the reproducibility of results and long term stability.

Quantum dots that allow a chemo-resistive read-out of H_2_S concentrations are also attractive. Employing PbS QDs, the detection of hydrogen sulfide in a concentration range that lies between units and hundreds of ppm has been reported with a lower detection limit of about 2 ppb [71,72]. The best response and recovery times reported are about twenty and two hundred seconds, respectively, and the optimal operating temperature is 135 °C. Even though the effect of ambient humidity has not been checked, cross-sensitivity to sulfur dioxide, nitrogen dioxide, or ammonia is low. H_2_S inducing p-to-n transition of PbS QDs has been suggested as the sensing mechanism. The fact that colloidal QDs are solution-processed nanomaterials is very attractive for keeping the fabrication costs of these sensors very low. However, further research would be necessary to fully validate this approach.

Some applications for hydrogen sulfide detection would require sensors to operate under harsh conditions such as low oxygen content or high temperature environments. This can be the case of H_2_S detection in petrochemical or power plants and in oil drilling facilities. Although none of the reviewed papers specifically discuss performance under harsh conditions, it can be anticipated that those sensors relying on the sulfurization of copper oxides upon exposure to hydrogen sulfide are good candidates for detecting this species with low oxygen content. This is because in this approach, the main sensing mechanism does not imply a change in the amount of the surface oxygen species. Indeed, some authors have already reported stable gas sensing responses employing metal oxides at trace level oxygen concentrations [23,78]. Recently, a few authors have reported SiC based materials for detecting gases at high temperatures [79,80,81], however, selective H_2_S detection at high temperatures has not been reported so far.

The results achieved by employing carbon nanomaterials show that these have potential for the detection of hydrogen sulfide. In most cases, fair responsiveness to H_2_S is reported at room temperature, which is of interest for wearable, battery operated detectors [56,57,61,62,64,65,66]. While most papers show hydrogen sulfide responses at ppm levels, Chen and co-workers [62] report a limit of detection of 1 ppb, with response and recovery times of a few minutes and tens of minutes, respectively. However, sensors employing carbon nanomaterials still present important drawbacks including lack of baseline recovery, poor stability, and important cross-sensitivity (to ambient moisture, ammonia, or nitrogen dioxide). Furthermore, in graphene based materials, many of the currently employed synthesis or preparation methods are not industrially scalable and, therefore, are not suitable for the mass production of H_2_S sensors.

## 6. Conclusions and Outlook

The last few years have seen a systematic increase in the demand for nanomaterials to be integrated in inexpensive, widespread gas detectors or gas sensing networks with superior performance and low power consumption. Here we have reviewed the advances for detecting hydrogen sulfide in the environment and identified the materials and methods that have reached maturity or that look more promising. In particular, it has been shown that iron oxide can be used for selectively detecting H_2_S, thanks to the chemiluminescence resulting from the catalytic oxidation of hydrogen sulfide onto this oxide. In addition, hybrid metal oxides containing cupric or cuprous oxides are excellent candidates for the selective detection of hydrogen sulfide, thanks to the formation of copper sulfides upon interaction with H_2_S. This constitutes one of the very few examples in which the selective detection of a target gas seems possible by employing uncomplicated, metal oxide chemo resistors. By further exploiting nanotechnology, it can be expected that more sensitive (thanks to higher specific surface area) and more stable (e.g., via better control of crystallinity and surface defects) metal oxides will be made available, which will operate at lower temperatures (even at room temperature). Additionally, the need of sensors that are able to reliably detect hydrogen sulfide under harsh conditions (e.g., low oxygen background or high temperatures) is likely to fuel the quest for metal oxides and silicon carbide hybrid materials. Organic-inorganic hybrid nanomaterials, especially those employing carbon nanomaterials, show potential for detecting hydrogen sulfide, but substantial research efforts are needed for these to reach a stage of maturity to be considered as valid alternatives. While one of their main advantages is the room-temperature responsiveness to H_2_S, efforts should be directed towards increasing selectivity, ameliorating stability, and reproducibility. Concerning the last issue, there is still a need for developing synthesis and functionalization techniques, especially in graphene, graphene oxide, and reduced graphene oxide materials, that enable the mass production of nanomaterials with highly reproducible properties. Finally, colloidal quantum dots also seem to be an interesting alternative for developing sensitive and remarkably selective chemo resistors for detecting hydrogen sulfide at low operating temperatures. These are solution-processed nanomaterials and, therefore, are suitable for the mass production of a new generation of inexpensive sensors on flexible substrates. However, the results reported remain preliminary and the use of lead sulfur QDs may represent a threat to the environment, therefore research on more environmentally-friendly materials should be considered to further develop this approach.

## Figures and Tables

**Table 1:** Summary of the main properties and performance characteristics of the different nanomaterials employed in the detection of H_2_S gas.

Nanomaterial	Transduction Scheme	Operating Temperature	Response to H_2_S	Range and LOD	Response Time	Recovery Time	Selectivity (Not Affected by, Unless Otherwise Indicated)	Reference
α-Fe_2_O_3_ film	Chemi luminescence	320 °C	1827 (100 ppm) ^1^	8–2000 ppm/3 ppm	15 s	120 s	Ethanol, hexane, cyclohexane, ethylene, hydrogen, o-dichlorobenzene, carbon dioxide, nitrogen dioxide, ammonia, thiophene, sulfur dioxide, dimethyl sulfur. Effect of ambient moisture not available.	[12]
SnO_2_ nanospheres	Chemi luminescence	160 °C	2000 (5 ppm) ^1^	NA ^5^	4 s	20 s	Carbon monoxide, hydrogen, nitrogen dioxide. Effect of ambient moisture not available.	[13]
α-Fe_2_O_3_ nanochains	Chemo resistor	285 °C	20 (100 ppm) ^2^	1–100 ppm/1 ppm	8.6 s	66 s	NA ^5^	[14]
α-Fe_2_O_3_ nanoparticles	Chemo resistor	300 °C	5 (10 ppm) ^2^	0.05–100 ppm/50 ppb	30 s	5 s	NA ^5^	[15]
Pt-doped α-Fe_2_O_3_ film	Chemo resistor	160 °C	330 (100 ppm) ^2^	10–1000 ppm/units of ppm	>1 min	>1 min	Ethane, carbon monoxide, significant cross-sensitivity to ethanol and ammonia. Effect of ambient moisture not available.	[16]
Fe-doped SnO_2_	Chemo resistor	150 °C	90 (100 ppm) ^2^	10–250 ppm/10 ppm	Few s	100 s	Carbon monoxide, liquefied petroleum gas, significant cross-sensitivity to ethanol. Effect of ambient moisture not available.	[18]
Fe_2_(MoO_4_)_3_ nanorods	Chemo resistor	80 °C	18 (50 ppm) ^2^	1–50 ppm/1 ppm	30 s	150 s	NA ^5^	[19]
NiFe_2_O_4_	Chemo resistor	300 °C	35.8 (5 ppm) ^2^	5–200 ppm/1 ppm	15 s	35 s	Liquefied petroleum gas, methane, carbon monoxide, butane, significant cross-sensitivity to hydrogen. Effect of ambient moisture not available.	[20]
Ni_0.6_Zn_0.4_Fe_2_O_4_	Chemo resistor	225 °C	0.8 (50 ppm) ^3^	NA ^5^	10 s	95 s	Significant cross-sensitivity to ethanol, liquefied petroleum gas, ammonia. Effect of ambient moisture not available.	[21]
Pure and metal loaded WO_3_	Chemo resistor	200 °C to 350 °C	25–190 (10 ppm) ^2^	0.2–200 ppm/200 ppb	1 s	11 min	Significant cross-sensitivity to ammonia, nitrogen dioxide and ambient moisture.	[22,23,24,25]
Mesoporous WO_3_	Chemo resistor	250 °C	325 (100 ppm) ^2^	0.25–200 ppm/250 ppb	2 s	38 s	Hydrogen, benzene, with some cross-sensitivity to methanol, ethanol, acetone, ammonia, and acetaldehyde. Effect of ambient moisture not available.	[33]
Pure or In doped ZnO	Chemo resistor	RT ^4^ to 250 °C	17 to 90 (100 ppm) ^2^	0.5 to 100 ppm/500 ppb	>20 min (RT); 2 s (250 °C)	>20 min (RT); 4 min (250 °C)	High cross-sensitivity to ammonia. Effect of ambient moisture not available.	[26,27,28]
ZnO nanoparticles	Shift of SPR peak	RT ^4^	0.71 nm/ppm ^6^	10 to 100 ppm/NA ^5^	1 min	1 min	Hydrogen, methane, ammonia, chlorine. Effect of ambient moisture not available.	[54]
ZnO nanorods	Lossy mode resonance	RT ^4^	0.8 nm/ppm ^6^	10 to 100 ppm/NA ^5^	NA ^5^	NA ^5^	Hydrogen, methane, ammonia, chlorine. Effect of ambient moisture not available. Response saturates at 60 ppm of H_2_S.	[55]
In_2_O_3_	Chemo resistor	270 °C	120 (50 ppm) ^2^	NA ^5^	NA ^5^	NA ^5^	Hydrogen, ammonia, toluene, benzene, carbon monoxide, methane. Significant cross-sensitivity to liquefied petroleum gas, formaldehyde, and ethanol. Important cross-sensitivity to nitrogen dioxide. Effect of ambient moisture not available.	[29,34]
CeO_2_	Chemo resistor	RT ^4^	2 (100 ppm) ^2^	0.1 to 100 ppm/100 ppb	20 s	200 s	Hydrogen, with some cross-sensitivity to ethanol and ammonia. Effect of ambient moisture not available.	[30]
YMnO_3_	Chemo resistor	100 °C	90 (500 ppm) ^2^	20 to 100 ppm/NA ^5^	6 s	6 s	Significant cross-sensitivity to liquefied petroleum gas, carbon monoxide, and hydrogen. Effect of ambient moisture not available. Response saturates for H_2_S concentrations higher than 100 ppm.	[31]
CuO-SnO_2_	Chemo resistor	200 °C	0.88 (100 ppm) ^3^	100 to 500 ppm/NA ^5^	60 s	40 s	NA ^5^. Response saturates for H_2_S concentrations higher than 300 ppm.	[35]
CuO-SnO_2_	Chemo resistor	300 °C	88 (2 ppm) ^2^	20 to 1000 ppb/20 ppb	10 min	10 min	NA ^5^	[36]
CuO-SnO_2_	Chemo resistor	160 °C	250 (100 ppm) ^2^	25 to 300 ppm/NA ^5^	10 min	NA ^5^	NA ^5^	[37]
CuO-SnO_2_	Chemo resistor	140 °C	10^6^ (10 ppm) ^2^	10 to 160 ppm/NA ^5^	2 min	>20 min	NA ^5^	[38]
CuO-SnO_2_	Chemo resistor	150 °C	2000 (20 ppm) ^2^	NA ^5^	14 s	21 s	NA ^5^	[39]
CuO-SnO_2_ nanoribbons	Chemo resistor	RT ^4^	1.8 × 10^4^ (3 ppm) ^2^	NA ^5^	15 s	>20 min	NA ^5^	[40]
CuO-SnO_2_	Chemo resistor	150 °C	0.81 (1000 ppm) ^3^	200–2500 ppm/tens of ppm	53 s	83 s	Hydrogen, carbon monoxide, liquefied petroleum gas, sulfur dioxide. Effect of ambient moisture not available. Response saturates for H_2_S concentrations higher than 1500 ppm.	[41]
CuO-SnO_2_ coral-like	Chemo resistor	100 °C	4173 (10 ppm) ^2^	0.1–10 ppm/20 ppb	10 s	>30 min	Ethanol, formaldehyde. Effect of ambient moisture not available.	[42]
Cu_2_O-SnO_2_	Chemo resistor	RT ^4^	0.6 (100 ppm) ^3^	5–150 ppm/1 ppm	>1 min	>1 min	Toluene. Significant cross-sensitivity to hydrogen liquefied petroleum gas, nitric oxide, ammonia. Effect of ambient moisture not available.	[43]
CuO-ZnO	Chemo resistor	225 °C	380 (10 ppm) ^2^	0.1–20 ppm/100 ppb	10 s	200 s	Ethanol, acetone, hydrogen, nitrogen dioxide, sulfur dioxide, methane, acetaldehyde. Effect of ambient moisture not available.	[44]
CuO-ZnO	Chemo resistor	200 °C	83.5 (5 ppm) ^2^	5–100 ppm/1 ppm	572 s	65 s	Carbon monoxide, ammonia, hydrogen, methane. Effect of ambient moisture not available.	[45]
Cu-ZnO	Shift of SPR peak	RT ^4^	0.2 nm/ppm ^6^	10–100 ppm/NA ^5^	1 min	1 min	NA ^5^	[52]
Cu-SnO_2_-ZnO	Chemo resistor	150 °C	6.4 × 10^4^ *	NA ^5^	NA ^5^	NA ^5^	Liquefied petroleum gas, carbon dioxide, nitrogen oxides, methane. Significant cross-sensitivity to carbon monoxide. Effect of ambient moisture not available.	[46]
Copper oxides-WO_3_	Chemo resistor	390 °C	26 (5 ppm) ^2^	0.3–5 ppm/100 ppb	2 s	684 s	Hydrogen, carbon monoxide, ammonia, benzene, nitrogen dioxide. Resilient to changes in the background humidity.	[47]
CuO nanosheets	Chemo resistor	100 °C	320 (1 ppm) ^2^	30–1200 ppb/20 ppb	10 s	10 s	Ammonia, carbon monoxide, nitrogen oxides, hydrogen. Strong cross-sensitivity to ambient moisture.	[48]
CuO	Chemo resistor	Switching 150–450 °C	1000 (5 ppm) ^2^	NA ^5^	<1 min	<1 min	Sulfur dioxide, nitrogen dioxide, ammonia. Slightly affected by changes in ambient moisture.	[50]
Au-TiO_2_-NiO	Absorbance change	350 °C	0.97 (100 ppm) ^7^	2–100 ppm/NA ^5^	1 min	7 min	Carbon monoxide, hydrogen. Effect of ambient moisture not available. Response signal saturates for H_2_S concentrations higher than 10 ppm.	[51]
Ag-NiO-ITO	Shift of SPR peak	RT ^4^	1 nm/ppm ^6^	10–100 ppm/NA ^5^	NA ^5^	NA ^5^	Hydrogen, ammonia, chlorine, carbon monoxide. Effect of ambient moisture not available.	[53]
Ag-SWCNT	Chemo resistor	RT ^4^	NA ^5^	NA ^5^	NA ^5^	NA ^5^	Cross-sensitivity to ammonia and nitric oxide. Effect of ambient moisture not available. Unstable response. Lack of baseline recovery.	[56]
Au-SWCNT	Chemo resistor	RT ^4^	0.23 (1 ppm) ^3^	20–1000 ppb/20 ppb	5 min	>20 min	NA ^5^. Response saturates at 250 ppb of H_2_S.	[57]
Co_3_O_4_–SWCNT	Chemo resistor	250 °C	5 (100 ppm) ^3^	10–150 ppm/units of ppm	2 min	10 min	Ammonia, methane, hydrogen. Effect of ambient moisture not available.	[59]
Cu-MWCNT	SAW	150 °C	240 kHz (100 ppm) ^8^	5–150 ppm/units of ppm	7 s	9 s	Hydrogen, ethanol, acetone. Ambient moisture affects the response (20% decrease in response at 150 °C).	[60]
Cu-Diamond Like Carbon	Shift of SPR peak	RT ^4^	NA ^5^	NA ^5^	1 min	NA ^5^	NA ^5^	[61]
Cu_2_O-Graphene	Chemo resistor	RT ^4^	0.35 (100 ppb) ^3^	5–100 ppb/1 ppb	2 min	10 min	Hydrogen, methane, ammonia. Significant cross-sensitivity to ammonia. Effect of ambient moisture not available.	[62]
MoO_3_–rGO	Chemo resistor	160 °C	4120 (50 ppm) ^2^	50–500 ppm/50 ppm	1 min	2 min	Ethanol, nitric oxide, carbon monoxide. Effect of ambient moisture not available.	[63]
PSS-doped PANI/graphene	Chemo resistor	RT ^4^	0.79 (50 ppm) ^3^	1–50 ppm/1 ppm	5 min	>20 min	Ethanol. Significant cross-sensitivity to ammonia. Effect of ambient moisture not available.	[64]
DDA-GO or EDA-GO or AMIDE-GO	Chemo resistor	RT ^4^	160 (50 ppm) ^2^	50–500 ppm/50 ppm	1 min	3 min	Carbon monoxide, nitric oxide. Significant cross-sensitivity to ethanol. Effect of ambient moisture not available.	[65,66]
Cu-Phthalo cynanine	ChemFET	RT ^4^	0.605 (200 ppm) ^9^	100–500 ppm/NA ^3^	89 s	290 s	Methane, hydrogen, carbon monoxide. Significant cross-sensitivity to ammonia. Effect of ambient moisture not available. Poor recovery of the baseline.	[67]
Polytiophene-WO_3_	Chemo resistor	70 °C	12 (100 ppm) ^2^	5–200 ppm/3 ppm	10 s	5 s	Methanol, ethanol, acetone, ammonia. Effect of ambient moisture not available.	[70]
Colloidal PbS QDs	Chemo resistor	135 °C	4218 (50 ppm) ^2^	5–100 ppm/6 ppb	23 s	171 s	Sulfur dioxide, nitrogen dioxide, ammonia. Effect of ambient moisture not available.	[71,72]
MOFs	Chemi luminescence	250 °C	7500 (16 ppm) ^1^	6–16 ppm/1 ppm	1 s	5 s	Methanol, ethanol, propanol, butanol, isobutanol, acetone, formaldehyde, toluene, chloroform. Effect of ambient moisture not available.	[73]

^1^ Chemoluminescence signal; ^2^ R_air_/R_gas_; ^3^ (R_air_ − R_gas_)/R_air_; ^4^ RT: Room temperature; ^5^ NA: Not available; ^6^ Shift in the SPR frequency; ^7^ Abs_gas_/Abs_air_; ^8^ Frequency shift; ^9^ (I_gas_ − I_air_)/I_air_; * Not clear how this sensitivity value is obtained.
